# Cranio-cervical hyperpneumatization: a case report

**DOI:** 10.1093/bjrcr/uaaf003

**Published:** 2025-01-23

**Authors:** Matthew Kueh, Ramnik Behar

**Affiliations:** Department of Radiology, Queen Alexandra Hospital, Portsmouth PO6 3LY, United Kingdom; Department of Radiology, Lymington New Forest Hospital, Lymington SO41 8QD, United Kingdom

**Keywords:** Hyperpneumatisation, atlanto-occipital, pneumorrhacis, epidural, aquired, valsalva, neuroradiology

## Abstract

Hyperpneumatization is a rare pathological process where air-filled cavitation form within solid bone architecture occurring at sites where physiological pneumatization is not seen. Extension of this process into the atlanto-occipital region is considered extremely rare and is only quoted several times in the literature. In this case report, we present a 66-year-old man who presented with an 8-month history of a worsening frontal headache and blocked sensation in his left ear. Subsequent CT head evaluation revealed hyperpneumatization affecting C1 vertebra, temporal and occipital bones with extension into the clivus. A rare complication of epidural emphysema was seen. The aetiology of hyperpneumatization is uncertain, although it is thought to be either congenital or acquired. In our case, clinical suggestion of eustachian tube dysfunction and radiological findings of thickened sinus mucosa and a unilateral nasal polyp point to chronic recurrent coryzal illnesses, which may indicate an acquired mechanism. Management is mostly conservative with surgical management reserved for high risk or refractory cases.

## Introduction

Pneumatization is a process where a solid bone develops air-filled cavities within its architecture. The process itself can be physiological and occurs from the final stages of foetal development through to early adulthood. Mastoid air cell development throughout childhood ending just after puberty is a classic example. Hyperpneumatization on the other hand, is a rare pathological process occurring at sites where physiological pneumatization is not seen. The aetiology of this is uncertain and is thought to either be congenital or acquired.

## Case report

A 66-year-old man presented to his GP with severe gripping frontal headaches occurring intermittently over 9 months which had been becoming more frequent over the last 3 months. For the last 40 years, he has been experiencing increased pressure in his left ear after swimming in the tropics and has been performing the Valsalva manoeuvre to relive his symptoms. A CT head scan was requested to look for any space occupying lesions.

**Figure 1.**
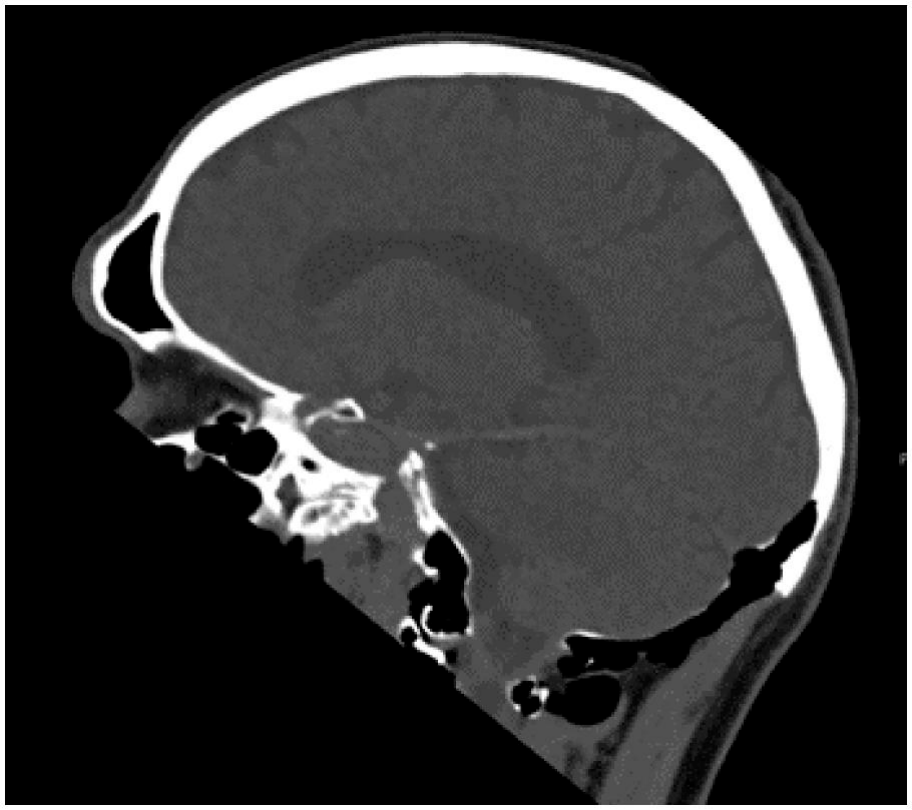
Pneumatization extending to atlas.

**Figure 2.**
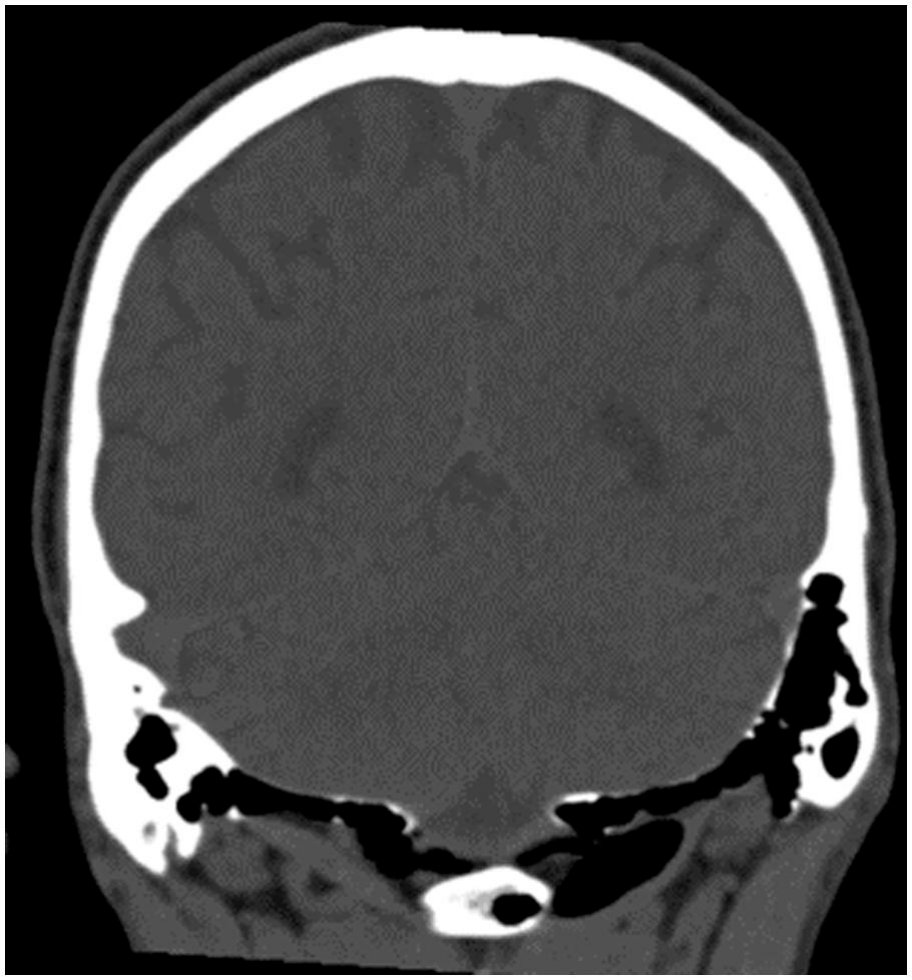
Pneumatization of posterior arch of atlas.

**Figure 3.**
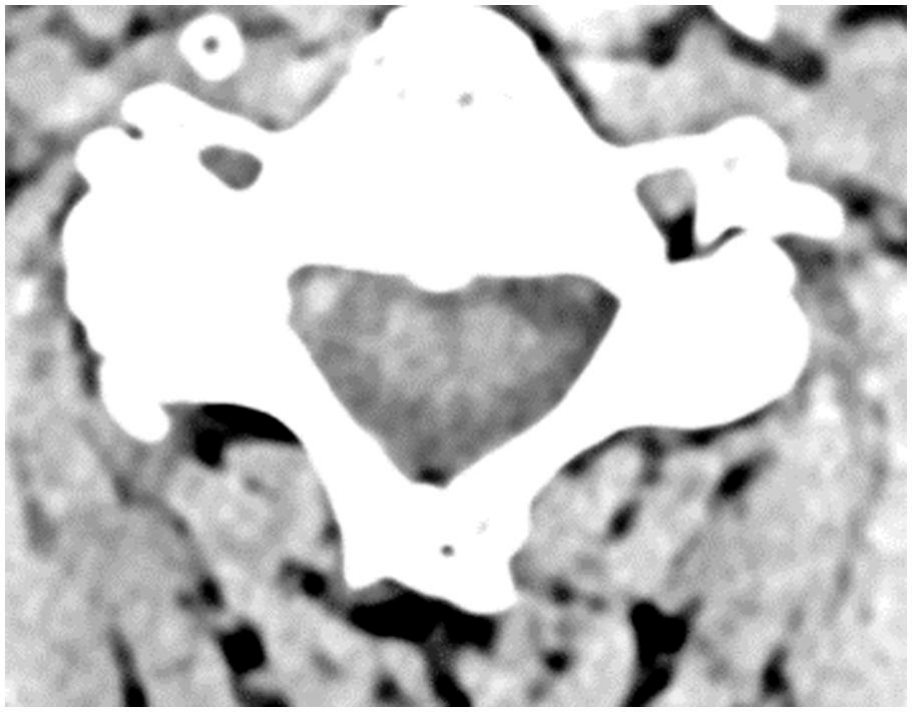
Subtle pneumorrhachis.

**Figure 4.**
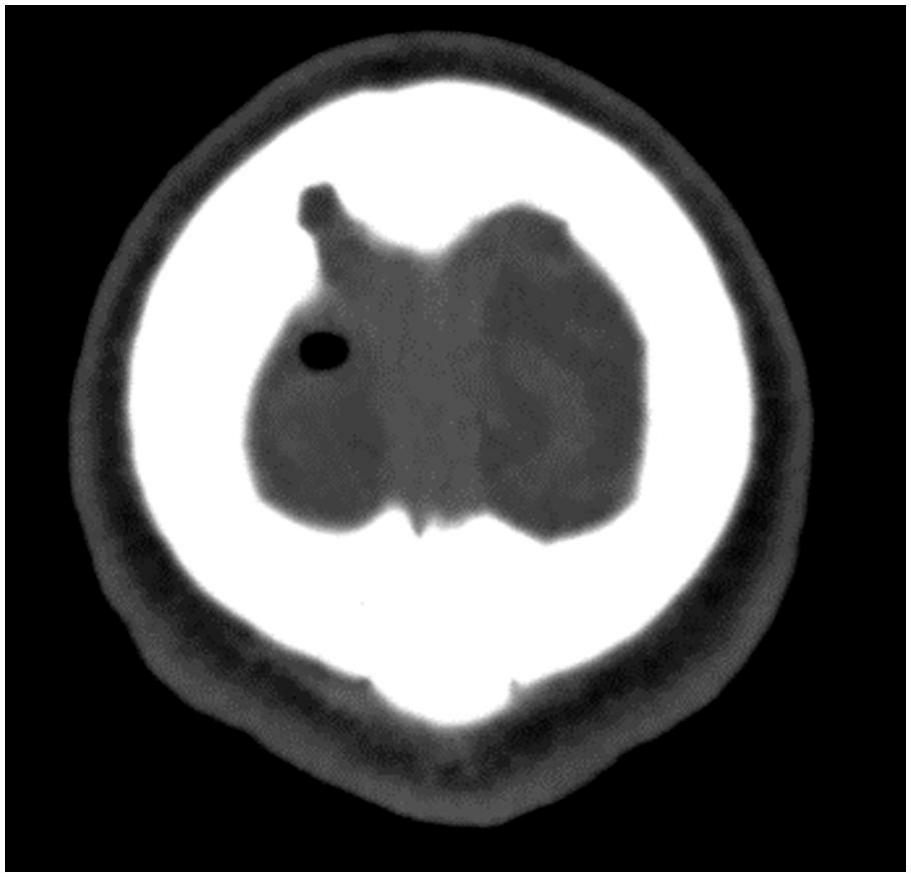
Epidural emphysema at the vertex.

The CT head demonstrated extensive craniocervical hyperpneumatization involving the C1 vertebra, temporal, and occipital bones with extension into the clivus (Figures 1 and 2), and pneumorrhacis within the spinal canal (Figure 3) and the epidural space at the vertex (Figure 4). There was also air in the soft tissues of the craniocervical junction in the posterior paraspinal musculature and prevertebral space. The patient was subsequently referred to a spinal multi-disciplinary team (MDT) who recommended follow-up imaging in the form of a CT venogram to rule out sinus stenosis.

Follow-up CT cervical spine demonstrated hyperpneumatization had not affected the rest of the cervical spine. A CT sinuses showed mucosal thickening within the frontal sinuses and ethmoid air cell, as well as a unilateral right polyp.

Given the history and imaging findings, idiopathic hyperpneumatization is the main differential. However, other differentials to consider would be a gas forming infection, lytic bone metastasis, or multiple myeloma.

## Discussion

Patients with cranio-cervical hyperpneumatization are often asymptomatic but when symptomatic the three main presenting symptoms are headaches, tinnitus, and posterior cervicalgia.^1^ Hence, the patient in this case presented typically. That being said, what was demonstrated on imaging was quite remarkable, given the extension of the hyperpneumatization from the temporal bones to the atlanto-occipital region which is extremely rare.

Complications in the literature include subcutaneous emphysema and pathological fractures.^1^ In our case, there was evidence of epidural emphysema or pneumorrhachis (Figures 3 and 4) which is a rare complication of hyperpneumatization.^1^^,^^2^

It is postulated that in the case of congenital causes of hyperpneumatization, low mesenchymal tissue production and incomplete closure of cranial sutures enable air to cross over cranial sutures and synovial joints more readily.^1^^,^^3^ The acquired theory hypothesizes that repetitive Valsalva manoeuvres lead to increased pressure in the middle ear thereby forcing air through the opening of the occipitomastoidal suture.^1^ In our patient, symptoms of fullness in his ear prior to presentation may indicate some form of eustachian tube dysfunction that prompted the repeated use of the Valsalva manoeuvre. Thickening of the sinus mucosa and unilateral nasal polyp may also indicate a history of repeated coryzal illness.

Conservative management of hyperpneumatization includes behavioural modification focused on reducing Valsalva manoeuvres and utilizing fewer damaging methods of equalizing middle ear pressure like yawning or chewing.^4^^,^^5^ These patients are also discouraged from engaging in high-risk activities that may result in pathological fractures.^4^ Surgical intervention can come in the form of external fixation, myringotomy, tympanostomy tube insertion, or mastoidectomy.^4^ In addition to the above, pneumorrhachis typically tends to resolve spontaneously; however, the presence of epidural emphysema would warrant antibiotics as there is likely a communication with the external environment.^4^

## Conclusion

Cranio-cervical hyperpneumatization is a rare condition with an uncertain aetiology where solid bone has pathologically developed air-filled cavities within its architecture. Our case is that of an even rarer subtype which involves the atlanto-occipital region with epidural emphysema.

## Learning points

- There is further evidence to suggest that the cause of hyperpneumatization is of an acquired aetiology in this case because of repeated Valsalva manoeuvres.
- Patients with eustachian tube dysfunction or engaging in high pressure activities should be aware of the risk of performing Valsalva manoeuvres excessively.
- Other less damaging techniques to equalize middle ear pressure such as yawning or chewing and the usage of decongestants and antihistamines to reduce inflammation of the eustachian tube should be suggested as alternatives to the Valsalva manoeuvre in patients with eustachian tube dysfunction.
- Epidural emphysema because of hyperpneumatization tends to resolve spontaneously and does not typically require intervention other than antibiotic cover.

## References

[1] AbdulAzeez MM , Huber P-Z, Alsaadi SB, Vladimir C-NB, Salazar LRM, Hoz SS. Cranio-cervical bone hyperpneumatization: an overview and illustrative case. J Acute Dis. 2018;7:145-148. 10.4103/2221-6189.241007

[2] Germino JC , Medverd JR, Nguyen VT, et al ‘Craniocervical hyperpneumatization with concurrent pneumorrhachis, pneumomediastinum, and subcutaneous emphysema in a weightlifter’. Spine J. 2013;13:e47-e53. 10.1016/j.spinee.2013.06.033 24095100

[3] Rebol J , Munda A, Tos M. Hyperpneumatization of the temporal, occipital and parietal bones. Eur Arch Otorhinolaryngol. 2004;261:445-448. 10.1007/s00405-003-0716-6 14652772

[4] Maccarrone F , Alicandri-Ciufelli M, Martone A, et al ‘Cranio-cervical hyperpneumatization: management of a complicated case’. Otorhinolaryngology. 2023;73:212-217. 10.23736/s2724-6302.23.02509-4

[5] Llewellyn A , Norman G, Harden M, et al *Interventions for Adult Eustachian Tube Dysfunction: A Systematic Review*. Southampton (UK): NIHR Journals Library; 2014. (Health Technology Assessment, No. 18.46.) Chapter 1, Background. 10.3310/hta18460 PMC4781384 25029951

